# Mild hypothermia during the reperfusion phase protects mitochondrial bioenergetics against ischemia-reperfusion injury in an animal model of ex-vivo liver transplantation—an experimental study

**DOI:** 10.7150/ijms.34617

**Published:** 2019-09-07

**Authors:** Rui Miguel Martins, João Soeiro Teodoro, Emanuel Furtado, Rui Caetano Oliveira, José Guilherme Tralhão, Anabela Pinto Rolo, Carlos Marques Palmeira

**Affiliations:** 1 Department of Surgery, Instituto Português de Oncologia de Coimbra, Coimbra, Portugal; 2 Department of Life Sciences, Faculty of Sciences and Technology, University of Coimbra; and Center of Neurosciences and Cell Biology, University of Coimbra, Coimbra, Portugal; 3 Unidade de Transplantação Hepática de Crianças e Adultos, Hospitais da Universidade de Coimbra, Centro Hospitalar e Universitário de Coimbra, Coimbra, Portugal; 4 Department of Pathology, Centro Hospitalar e Universitário de Coimbra, Coimbra, Portugal; 5 Department of Surgery, Hospitais da Universidade de Coimbra, Centro Hospitalar e Universitário de Coimbra, Coimbra, Portugal; Clínica Universitária de Cirurgia III, Faculty of Medicine, University of Coimbra, Coimbra, Portugal; and Center for Investigation on Environment, Genetics and Oncobiology (CIMAGO), Faculty of Medicine, University of Coimbra, Coimbra, Portugal

**Keywords:** hypothermia, mitochondria, bioenergetics, adenosine triphosphate, liver transplantation

## Abstract

The organ preservation paradigm has changed following the development of new ways to preserve organs. The use of machine perfusion to preserve organs appears to have several advantages compared with conventional static cold storage. For liver transplants, the temperature control provided by machine perfusion improves organ preservation. In this experimental study, we measured the effects of different temperatures on mitochondrial bioenergetics during the reperfusion phase. An experimental model of ex-vivo liver transplantation was developed in Wistar rats (*Rattus norvegicus*). After total hepatectomy, cold static preservation occurred at 4ºC and reperfusion was performed at 37ºC and 32ºC using a Langendorff system. We measured parameters associated with mitochondrial bioenergetics in the livers. Compared with the livers that underwent normothermic reperfusion, mild hypothermia during reperfusion caused significant increases in the mitochondrial membrane potential, the adenosine triphosphate content, and mitochondrial respiration, and a significant reduction in the lag phase (all *P* < 0.001). Mild hypothermia during reperfusion reduced the effect of ischemia-reperfusion injury on mitochondrial activity in liver tissue and promoted an increase in bioenergetic availability compared with normothermic reperfusion.

## Introduction

The lack of available organs is the principal limitation associated with liver transplantation. To increase the quantity of donor organs, marginal organs have been used, including those from elderly donors and patients with hepatic steatosis, those that have experienced prolonged cold ischemia, and those obtained after cardiac death 1, 2. The use of these poor-quality organs affects the clinical outcomes of liver transplantation, which has led to the development of new ways to preserve organs 3, 4. Ex-vivo machine perfusion of the liver is an alternative to conventional static cold storage, but there is no agreement about the most beneficial temperature 5, 6. Another issue that requires resolution is whether or not these liver preservation methods can be combined 7.

Machine perfusion is associated with declines in primary non-function, graft failure, and biliary complications. For liver ex-vivo preservation the standard of organ preservation has not established, contrary to the kidney ex-vivo preservation where the hypothermic perfusion has become the standard 8-10.

The process of cold and warm ischemia followed by a reperfusion period is specific to liver transplantation, and is the primary cause of cellular damage 11. Ischemia-reperfusion (I/R) injury compromises mitochondrial function and bioenergetics, particularly during reperfusion when the readmission of oxygen increases the production of reactive oxygen species 12, 13. We aimed to investigate mitochondrial function and cellular bioenergetics at different temperatures in an experimental model of ex-vivo liver transplantation, with a particular focus on the reperfusion phase.

## Materials and Methods

The materials and methods used in this study have been described in detail previously 13.

### Animals

Twelve-week-old male Wistar rats (*Rattus norvegicus*) weighing 320-350 g were purchased from Charles River (Charles River, Lyon, France). Upon arrival, the animals acclimatized for 1 week, and they were housed in an environment comprising controlled temperature and humidity and 12-h light-dark cycles, and given unlimited access to standard rodent food and acidified water. The study's protocol was approved by the Animal Ethics Committee at the University of Coimbra's Faculty of Medicine (ORBEA 150 2016/04112016, April 11, 2016). All of the studies were conducted in accordance with the principles and procedures in the EU (1986/609/EEC and 2010/63/EU), Federation of European Laboratory Animal Science Associations, and Animal Research: Reporting of In Vivo Experiments (ARRIVE) guidelines, and they were approved by the Animal Care Committee at the Center for Neurosciences and Cell Biology, University of Coimbra. We also applied the principles of the ARRIVE guidelines to data management and interpretation, and we minimized the number of animals used and their suffering.

### Chemicals and reagents

Except when noted, all of the chemicals and reagents were purchased from Sigma-Aldrich Corporation (St. Louis, MO, USA). All of the reagents and chemicals used were of the highest commercially available purity.

### Surgical protocol

**Figure 1:**
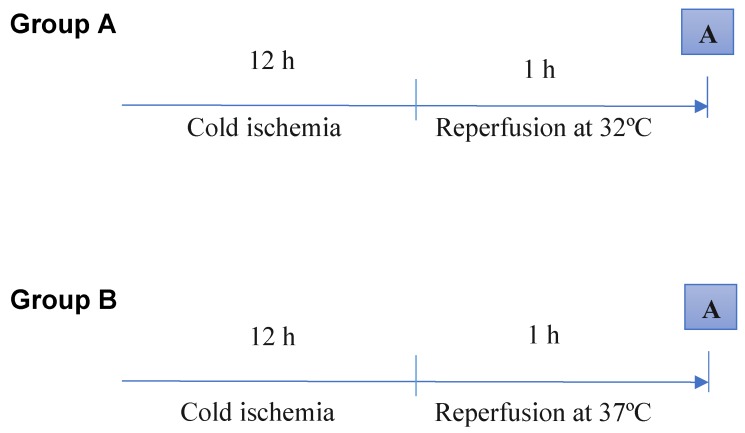
Schematic representation of reperfusion under hypothermic and normothermic conditions. Biopsies were taken at the end of the reperfusion time (A). The control group is not represented. Ten animals were analyzed per group.

The surgical procedures were performed under anesthesia induced by ketamine (50 mg/kg) and chlorpromazine (50 mg/kg), provided by the same operator. A median laparotomy was performed, and the liver was mobilized by dividing the hepatic ligaments. The experimental model of ex-vivo liver transplantation comprised the introduction of a cannula into the portal vein and hepatic perfusion with an organ preservation solution (Celsior^®^) at 4ºC for 10 min. Then, we performed a total hepatectomy while keeping the cannula inside the portal vein. Adequate inflows and outflows were confirmed. Cold static preservation at 4ºC was performed over 12 h. Reperfusion was performed using a Langendorff system at 32ºC or 37ºC for 1 h with a mixture comprising 50% Plasma-Lyte 148 and 50% Krebs solution at pH 7.2 that was supplemented with oxygen by a pressurized membrane oxygenator (pO2, 400-500 mm Hg) 14 (Fig. 1).

The animals (*n* = 30) were divided into three groups. The control group (*n* = 10) underwent a sham laparotomy, isolation of the hepatic pedicle, cannulation of the portal vein, perfusion with the organ preservation solution at 4ºC for 10 min, and total hepatectomy. Group A (*n* = 10) underwent a sham laparotomy, isolation of the hepatic pedicle, cannulation of the portal vein, perfusion with the organ preservation solution at 4ºC for 10 min, total hepatectomy, cold static preservation at 4ºC for 12 h, and reperfusion at 32ºC with the Plasma-Lyte/Krebs solution (pH 7.2) supplemented with oxygen for 1 h. Group B (n = 10) underwent a sham laparotomy, isolation of the hepatic pedicle, cannulation of the portal vein, perfusion with the organ preservation solution at 4ºC for 10 min, total hepatectomy, cold static preservation at 4ºC for 12 h, and reperfusion at 37ºC with the Plasma-Lyte/Krebs solution (pH 7.2) supplemented with oxygen for 1 h.

### Mitochondrial isolation

The mitochondria were isolated in a homogenization medium comprising 250 mM sucrose, 10 mM 4-(2-hydroxyethyl)-1-piperazineethanesulfonic acid (HEPES) (pH 7.4), 0.5 mM ethylene glycol-bis(β-aminoethyl ether)-N,N,N′,N′-tetraacetic acid (EGTA), and 0.1% fat-free bovine serum albumin (BSA) 15, 16. After homogenization of the minced blood-free hepatic tissue, the homogenates were centrifuged at 800 *g* for 10 min at 4°C. The supernatants were spun at 10 000 *g* for 10 min at 4°C to pellet the mitochondria that were then resuspended in a final washing medium from which EGTA and BSA were omitted, and it was adjusted to pH 7.4. The protein content was determined using the biuret method calibrated with BSA.

### Mitochondrial membrane potential measurements

The mitochondrial membrane potential was estimated using an ion-selective electrode to measure the distribution of tetraphenylphosphonium (TPP^+^). The voltage response of the TPP^+^ electrode to log (TPP^+^) was linear with a slope of 59 ± 1, and it conformed to the Nernst equation. The mitochondria (1 mg) were suspended in standard medium (1 mL), comprising 130 mM sucrose, 50 mM potassium chloride, 5 mM magnesium chloride, 5 mM monopotassium phosphate, 50 mM EDTA, 5 mM HEPES (pH 7.4), and 2 µM rotenone, supplemented with 3 µL TPP^+^. A matrix volume of 1.1 µL/mg protein was assumed. The reactions were carried out at 25°C in a temperature-controlled chamber surrounded by a water jacket with magnetic stirring. The membrane potential (mV), depolarization (mV), lag phase (s), and repolarization (mV) were measured, and the readings were recorded in triplicate.

### Oxygen consumption measurements

The oxygen consumption of the isolated mitochondria was determined using a Clark-type polarographic oxygen electrode (Oxygraph; Hansatech Instruments Ltd., King's Lynn, Norfolk, United Kingdom) 17. Mitochondria (1 mg) were suspended in the standard medium (1.4 mL) with constant stirring at 25°C, as described previously. The mitochondria were energized with succinate (5 mM) and state 3 respiration was induced by adding adenosine diphosphate (ADP) (200 nmol). Oxygen consumption was also measured in the presence of 1 µM carbonyl cyanide-p-trifluoromethoxyphenylhydrazone. State 3 respiration and the respiratory control ratio were calculated according to Chance and Williams 18.

### Adenosine triphosphate measurements

Liver adenosine triphosphate (ATP) was extracted using an alkaline extraction procedure 19. The tissue ATP levels were measured using a luciferase/luciferin assay kit and a PerkinElmer Victor 3™ plate-reader fluorometer (PerkinElmer, Waltham, MA, USA), according to the manufacturers' instructions.

### Histological analysis

The tissue samples were grossly inspected and divided, fixed in 4% formaldehyde, embedded in paraffin wax, cut into 4-µm sections, and stained with hematoxylin and eosin (Polysciences Inc., Warrington, PA, USA) using a Sakura Autostainer-Prisma 81D (Sakura Finetek Europe B.V., Alphen aan den Rijn, The Netherlands). An experienced pathologist who was blinded to the experimental groups, examined the tissue sections using a light microscope (Nikon Eclipse 50i; Nikon Corporation, Tokyo, Japan), and images were obtained using a Nikon-Digital Sight DS-Fi1 camera (Nikon Corporation).

### Statistical analysis

The continuous variables are presented as the means and standard errors of the means, unless otherwise specified. The normality of the data distributions was confirmed using the Kolmogorov-Smirnov and Shapiro-Wilk tests when indicated. Between-group comparisons were performed using Student's t-test, and differences among three or more groups were analyzed using a one-way analysis of variance for post hoc multiple comparisons. The statistical analyses were performed using IBM^®^SPSS^®^ software, version 22.0 (IBM Corporation, Armonk, NY, USA). A value of *P* < 0.05 was considered statistically significant.

## Results

Reperfusion under hypothermic conditions was performed to evaluate its effects on mitochondrial function and bioenergetics. In this study, the cold ischemia (12 h) and reperfusion (1 h) times were maintained. Reperfusion occurred at 32°C in group A and at 37°C in group B.

### Mitochondrial membrane potential

**Figure 2:**
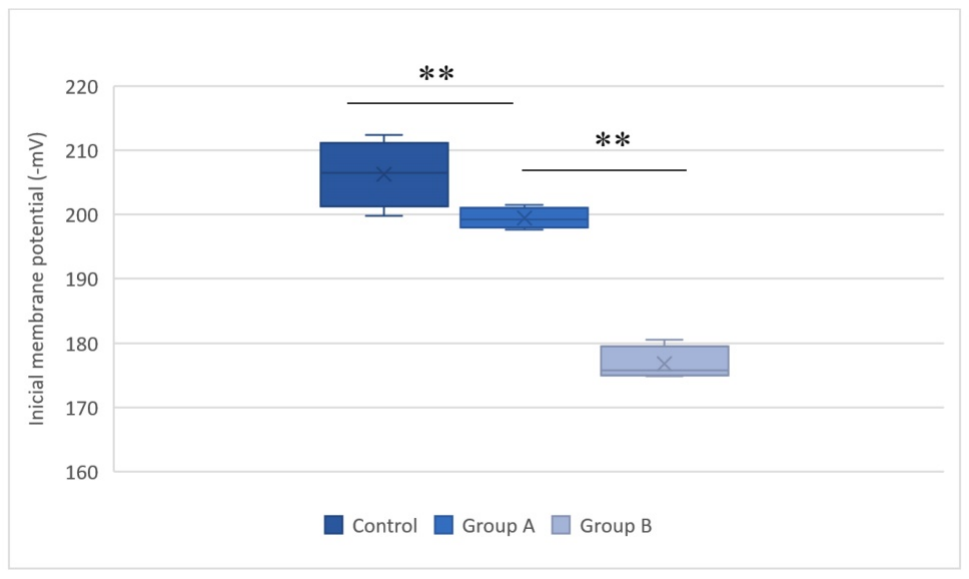
Initial membrane potentials (Δψ) in the control group, group A (hypothermic reperfusion), and group B (normothermic reperfusion). The membrane potentials were determined in the presence of succinate as a respiratory substrate. Phosphorylation was induced by adding adenosine diphosphate (100 nmol). A statistically significant difference was found between groups A (hypothermic reperfusion) and B (normothermic reperfusion). **P < 0.01.

**Figure 3:**
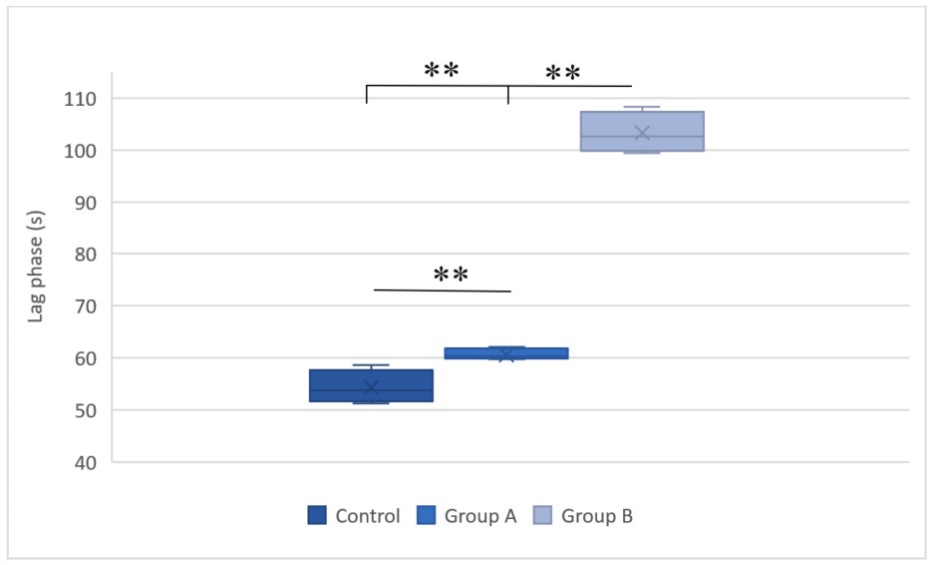
Lag phases in the control group, group A (hypothermic reperfusion), and group B (normothermic reperfusion) in the presence of succinate as a respiratory substrate. Phosphorylation was induced by adding adenosine diphosphate (100 nmol). A statistically significant difference was found between groups A (hypothermic reperfusion) and B (normothermic reperfusion). **P < 0.01.

The mitochondrial membrane potential estimates the phosphorylative capacity of isolated liver mitochondria. In this study, succinate was used to obtain the membrane potential data. A statistically significant difference in the mitochondrial membrane potential was evident between the groups (Table 1). Compared with the group subjected to normothermic reperfusion, hypothermic reperfusion significantly improved the parameters associated with mitochondrial function (*P* < 0.001). The lag phase declined in the hypothermic reperfusion group compared with that in the normothermic reperfusion group, thereby validating the measurement of the membrane potential data (Figs. 2 and 3).

### Mitochondrial respiration

**Figure 4:**
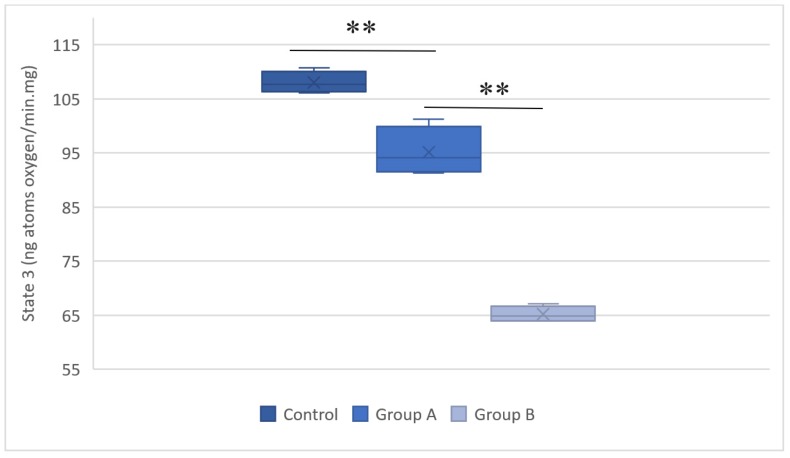
The respiratory state 3 values for the control group, group A (hypothermal reperfusion), and group B (normothermic reperfusion). The respiratory status was determined in the presence of succinate. A statistically significant difference was found between groups A (hypothermic reperfusion) and B (normothermic reperfusion). **P < 0.01.

**Figure 5:**
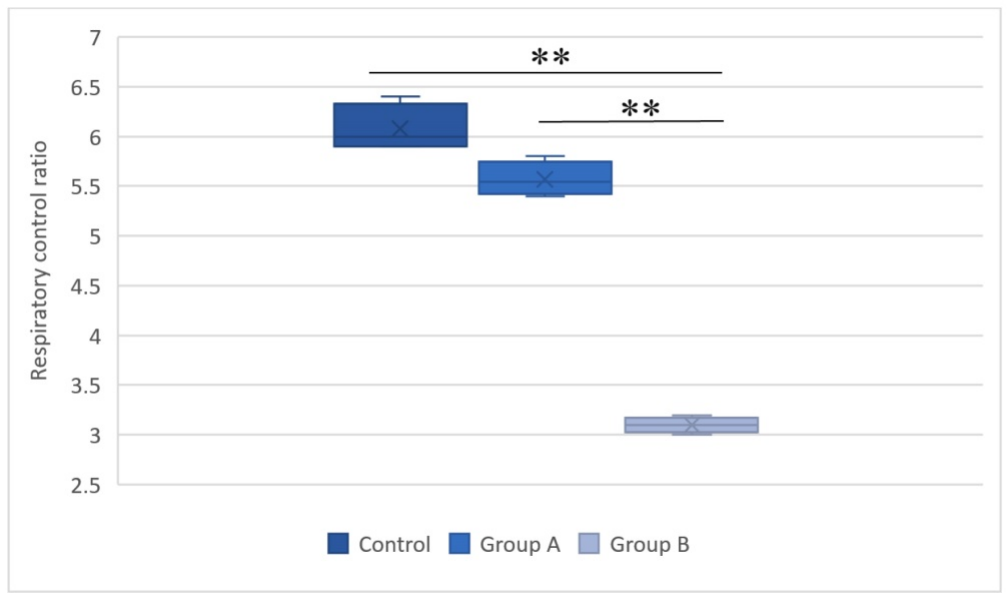
The respiratory control ratios in the control group, group A (hypothermal reperfusion), and group B (normothermic reperfusion). The respiratory control index was determined in the presence of succinate. A statistically significant difference was found between groups A (hypothermic reperfusion) and B (normothermic reperfusion). **P < 0.01.

The mitochondrial respiration measurements evaluated oxygen consumption after respiration was induced with succinate. Figures 4 and 5 summarize the results.

### Adenosine triphosphate content

**Figure 6:**
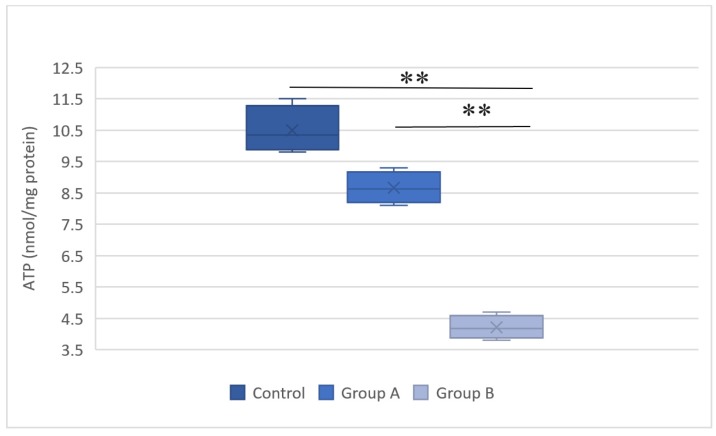
Representative plot of the adenosine triphosphate (ATP) levels in the hepatic tissue of the control group, group A (hypothermic reperfusion), and group B (normothermic reperfusion). A statistically significant difference was found between groups A (hypothermic reperfusion) and B (normothermic reperfusion). **P < 0.01. ATP, adenosine triphosphate.

Figure 6 illustrates the ATP levels in the hepatic tissue subjected to hypothermic and normothermic reperfusion. Lower ATP levels were present in the tissues subjected to normothermic reperfusion compared with those in the tissues subjected to hypothermic reperfusion.

### Histological evaluation

**Figure 7:**
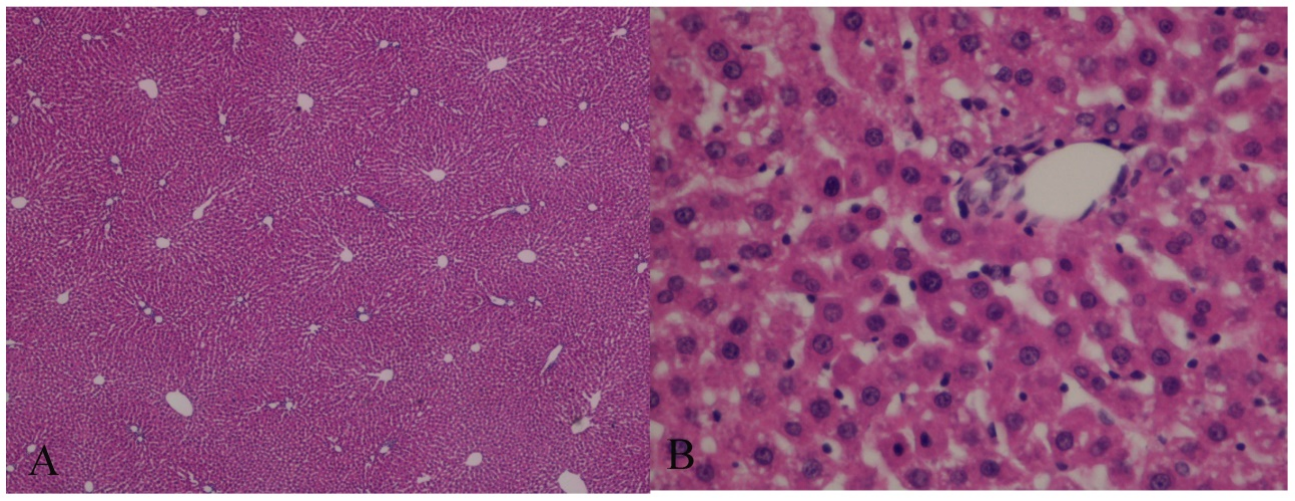
Hematoxylin and eosin (H&E)-stained sections of hepatic tissue from the hypothermic reperfusion group. The hepatic sinusoids do not show endothelial injury, and the hepatocytes contained normal intracellular organelles and nuclei, with no signs of apoptotic or necrosis (A: H&E 40×; B: H&E 400×).

**Figure 8:**
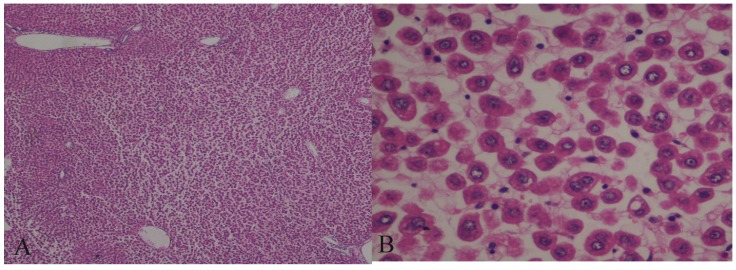
Hematoxylin and eosin (H&E)-stained sections of hepatic tissue from the normothermic reperfusion group. The hepatic parenchyma architecture is preserved without lesions. There is moderate-to-severe disassociation of the hepatocytes. The hepatocytes contain normal nuclei and organelles, with no signs of necrosis or apoptosis (A: H&E 40×; B: H&E 400×).

The histological evaluation of the hepatic tissue from the control group showed normal liver architecture. In Group A, the hepatic tissue was preserved, and there was no evidence of an inflammatory infiltrate, steatosis, or fibrosis. The structural integrity of the nuclei and organelles within the hepatocytes was maintained, and there was no evidence of necrosis or apoptosis. In Group B, the structure of the hepatic parenchyma was preserved, but the hepatocytes showed moderate-to-severe disassociation and some ballonization. The structural integrity of the organelles and nuclei within the hepatocytes was maintained, and neither apoptosis nor necrosis was visible (Figs. 7 and 8).

## Discussion

Animal models of hepatic transplantation are fundamental tools that have enhanced our understanding of the biological and immunological mechanisms involved in transplantation, and, thus, they have helped to answer some clinically relevant questions.

The mouse is the most commonly used animal, and several mouse models have been developed 20. In 1973, Lee et al. reported the first orthotopic liver transplantation in the rat, which consisted of performing an extracorporeal shunt between the portal and jugular vein at the recipient and posterior anastomosis of the implant to the hepatic vein, the portal vein and the recipient's aorta 21. This very complex technique was abandoned, and 18 years later, following the development of vascular microsurgery techniques, Qian *et al.* developed a complex animal model of orthotopic liver transplantation. However, the investigations based on this model are very limited, because it requires a high level of microsurgical expertise and specific technical conditions. In addition, the high mortality rate caused by disruptions to hepatocellular function has limited the use of this animal model 22, 23.

Oscar Langendorff evaluated physiological and pathophysiological events within ex-vivo heart tissue, and, consequently, other animal models were developed that enabled ex-vivo evaluations of the liver, with an emphasis on I/R studies 24-26. Previous ex-vivo liver transplantation studies are invaluable, because they have paved the way for the physiological and pathophysiological studies that are essential for the development of new ways to preserve the liver using dynamic preservation machines. These studies contribute to the development of dynamic preservation has altered the ways in which organs are perfused, preserved, and transported 5, 8, 27-30.

The animal models of ex-vivo liver transplantation are highly reproducible, and the results are not influenced by the complex surgical procedures of other models such as orthotopic liver transplant model. Despite these, the main limitation to these animal models of liver transplantation is related to the impossibility to evaluate pos-operative biomarkers of the liver function and the non-use of blood in the reperfusion phase 31.

Functional evaluations of mitochondrial activity in rodents have demonstrated that, like human beings, I/R clearly affects mitochondrial function, which has implications for bioenergetics, and translates into lower energy production efficiency 32, 33. This ATP deficiency is sufficient to trigger changes in cellular metabolism; therefore, I/R injury in liver transplants interferes with the cellular bioenergetic balance.

Bigelow *et al.* introduced the concept of hypothermia to clinical practice in the early 1950s, and they demonstrated its neuroprotective effect during cardiac surgery 34. The benefits of hypothermia include the preservation of hepatic metabolism, and reductions in the inflammatory response and apoptosis during ischemia 35. Recent experimental studies have shown that mild hypothermia at 32-34°C exerts a protective effect against warm I/R injury, but the mechanisms underlying this effect remain unclear 36. Azoulay *et al.* studied patients who underwent complex liver surgery as a consequence of central hepatic tumors involving the inferior vena cava or the confluence of the hepatic veins with the vena cava, and they demonstrated the protective effect of hypothermic in-situ hepatic perfusion compared with total vascular exclusion for >60 min. This study's findings showed that patients who underwent hypothermic perfusion had a better I/R-induced injury tolerance, which translated into improved postoperative liver and kidney function and reduced morbidity 37.

In this study, we undertook a laboratory evaluation of the concept of hypothermia applied to reperfusion during hepatic transplantation; this involved a reperfusion temperature of 32°C, which, according to experimental studies, provides more effective protection 38, 39. Our study's findings showed statistically significant differences between the hypothermic reperfusion group and the normothermic reperfusion group regarding the mitochondrial membrane potential and respiration parameters, which were preserved to higher degrees in the hypothermic reperfusion group. In addition, the amount of ATP produced in the hepatic tissue from the hypothermic reperfusion group was higher than that recorded in the hepatic tissue from the normothermic reperfusion group.

Compared with normothermic reperfusion, hypothermic reperfusion reduced the effect of I/R on mitochondrial activity, thereby increasing the bioenergetic availability (42%). Hence, applying hypothermic reperfusion to liver transplantation may be beneficial from a bioenergetic perspective, because mitochondrial function is preserved.

One of the main limitations regarding the use of hypothermia in clinical practice is the potential for coagulopathy. This seems to be associated with platelet dysfunction and damage to the enzymes in the coagulation cascade 40. The risk of bleeding and the subsequent need for transfusions increase by approximately 20% for each degree Celsius decline in the core temperature. Hypothermia reduces the metabolic rate by 8% for each degree Celsius decline 41, which, for this study, would imply a 34% reduction in metabolic activity. In humans, the only clinical applications of hypothermia that have led to improved outcomes are extra-hospital cardiac arrest and neonatal asphyxia 42, 43. To integrate the concept of hypothermic reperfusion into clinical practice and apply it to hepatic transplantation, further functional and technical studies will be necessary.

## Figures and Tables

**Table 1:** The membrane potentials and lag phases in the control group, group A (hypothermic reperfusion), and group B (normothermic reperfusion).

	Succinate		
	Control	Group A	Group B
**Initial membrane potential (-mV)**	207.4 ± 5.0	199.6 ± 1.5	176.4 ± 2.3**
**Depolarization (mV)**	24.0 ± 1.0	21.7 ± 1.1	16.9 ± 0.8**
**Lag phase (s)**	54.6 ± 2.8	60.8 ± 1.0	104.4 ± 4.1**
**Repolarization (mV)**	194.7 ± 7.7	189.8 ± 5.1	172.6 ± 2.1**

The data presented are the means and standard errors of the means. Statistically significant differences were found between groups A (hypothermic reperfusion) and B (normothermic reperfusion). ** *P* < 0.01.
